# Engineered bacteria and bacterial derivatives as advanced therapeutics for inflammatory bowel disease

**DOI:** 10.1042/EBC20253003

**Published:** 2025-02-27

**Authors:** Jingyuan Wu, Wanlin Ye, Jie Yu, Tuoyu Zhou, Nuo Zhou, Dennis K.P. Ng, Zhaoting Li

**Affiliations:** 1 School of Medicine, The Chinese University of Hong Kong, Shenzhen, Guangdong, 518172, P. R. China; 2 NMPA Key Laboratory for Research and Evaluation of Pharmaceutical Preparations and Excipients, State Key Laboratory of Natural Medicines, Department of Pharmaceutics, China Pharmaceutical University, Nanjing, 210009, P. R. China; 3 The Second Affiliated Hospital, School of Medicine, The Chinese University of Hong Kong, Shenzhen & Longgang District People’s Hospital of Shenzhen, Guangdong, 518172, P. R. China; 4 Department of Chemistry, The Chinese University of Hong Kong, Shatin, N. T., Hong Kong, P. R. China

**Keywords:** engineered bacteria, inflammatory bowel disease, synthetic biology, outer membrane vesicles, oral delivery system

## Abstract

Inflammatory bowel disease (IBD), a chronic and relapsing-remitting condition, is inadequately managed by conventional therapies that often lack targeting specificity and carry significant side effects, particularly failing to address intestinal barrier repair and microbial balance. Probiotics, with their strong colonization capabilities, present a novel approach to drug delivery. Various engineering strategies have been developed to enhance the targeting ability of probiotics to inflammation sites, enabling precise delivery or *in situ* synthesis of therapeutic molecules to expand their multifunctional potential. This review discusses the recent advancements in bacterial modifications, including surface physico-chemical and biological coating, genetic engineering, outer membrane vesicles, minicells, and bacterial ghosts, all of which can enhance therapeutic localization. We also outline critical preclinical considerations, such as delivery frequency, systemic distribution, immune evasion, and gene contamination risks, for clinical translation. These engineered bacteria and bacterial derivatives hold great promise for personalized and sustained IBD treatments, providing a new frontier for therapy tailored to the complex inflammatory environment of IBD.

## Introduction

Inflammatory bowel disease (IBD) is a long-lasting gastrointestinal condition that includes two subtypes, ulcerative colitis (UC) and Crohn’s disease (CD), both of which significantly raise the risk of colorectal cancer [1]. In the unique physiological progression of IBD, immune dysfunction manifested as the host response to its own tissues leads to aberrant excessive activation of the immune system. The gut microbiota dysbiosis further aggravates the intestinal inflammatory environment. Meanwhile, the impaired intestinal barrier function allows harmful substances to penetrate the mucosal barrier, enter the body, and provoke a stronger immune response. In addition, the excessive production of cytokines continuously amplifies the response and forms a vicious cycle. These inter-related factors drive the chronic progression and repeated attacks of IBD [2–4]. Traditional therapies mainly include 5-aminosalicylic acid, antibiotics (metronidazole and tinidazole), steroids (prednisone and hydrocortisone), immunosuppressants (methotrexate and cyclosporine), and biologics like infliximab and adalimumab [5,6]. The limited target specificity of traditional drugs means that they cannot accurately distinguish diseased tissues from normal parts, leading to unsatisfactory clinical usage. The short retention time means that their effective concentrations are not maintained for enough time, requiring frequent dosing. In addition, conventional drugs are accompanied by obvious systemic adverse reactions [7,8]. Furthermore, in the pathological IBD progression, the tricky problems of intestinal mucosal damage, leaky bowel syndromes, and gut microbiota dysregulation require more integrated approaches. The delicate design for drug delivery systems should improve the drug targeting specificity, extend the drug retention time, and reduce side effects, so as to improve the treatment experience of patients [9–11].

Different bacteria species play distinct roles in the gut, and their involvement in IBD can vary. Some bacteria may act as the causative pathogens of IBD, while others are viewed as beneficial and contribute positively to the treatment of the disease [12–14]. Through the modification and engineering of these probiotics, it is possible to regulate the balance of the intestinal microbiota, strengthen the intestinal barrier, and modulate the immune environment, thereby facilitating the therapeutic management of IBD [15]. Bacteria have prominent advantages as carriers in drug delivery systems. First, they proliferate rapidly and can, thus, maintain a high level of drug concentration in the targeted region. Secondly, some commensal bacteria have a strong survival ability to colonize, ensuring continuous drug release that can reduce the frequency of medication. Thirdly, by using bacteria as noninvasive oral delivery vectors, compared with injection and other invasive methods, the patient compliance is higher. Since IBD primarily affects the gastrointestinal tract, oral delivery of engineered bacteria and their derivatives offers natural advantages, such as high biosafety, good patient compliance, and the ability to regulate intestinal flora balance [16,17]. However, hurdles remain, particularly in bacterial colonization due to the harsh gastrointestinal environment, and in quality control to ensure consistent dosing and adherence to manufacturing practice [18]. More importantly, they can be genetically modified to achieve a responding release cycle, maintaining effective treatment with reduced discomfort [19,20]. Probiotics, in particular, hold great therapeutic potential for IBD, either for direct therapy or as drug delivery carriers [21]. Through chemical, physical, or biological modification of the surface of probiotics, it is possible to alter their composition and structural display to enhance their chemotaxis and colonization at inflammation sites in the intestines [22]. Synthetic biology involves the design and construction of biological systems to achieve specific functions. Utilizing synthetic biology tools and methodologies, engineered bacteria and their derivatives are genetically modified to improve their effectiveness in disease imaging, diagnosis, and treatment [23]. In particular, genetically engineered bacteria are central to this process, as they involve precise gene regulation, the design and construction of biological circuits, and improvements in safety and targeting capabilities, all of which are fundamental to the advancements of synthetic biology [24]. Progresses in synthetic biology have facilitated genetically engineered modifications that enable surface display and biosynthesis of molecules of interest specifically at the diseased sites [25]. In addition, the outer membrane vesicles (OMVs) from bacteria can effectively penetrate the intestinal epithelial cells to deliver agents to sites of inflammation and improve the genetic stability of wildtype strains [26,27]. Bacterial derivatives, including minicells and bacterial ghosts (BGs), can also encapsulate small molecular drugs and biologics, thus expanding the functional diversity of bacterial strains [28]. Therefore, oral delivery of engineered bacteria and bacterial derivatives provides promising opportunities for clinical treatment by exerting sustained and stable therapeutic effects in the unique, complex, and inflammatory environment of IBD.

**Figure 1:**
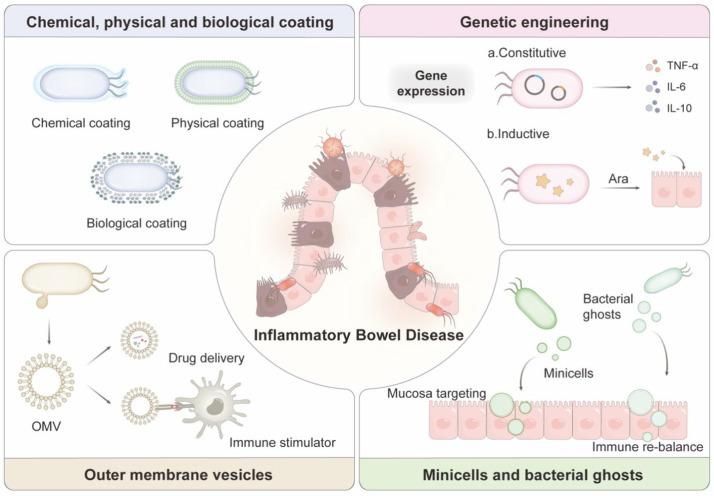
Schematic illustration of modification strategies in bacteria and bacterial derivatives in inflammatory bowel disease (IBD) treatment.

Despite the significant progress in preclinical research, the clinical application of engineered bacterial therapy as IBD therapeutics or drug delivery systems remains a major challenge. To maintain metabolic viability during large-scale transport and storage, an efficient and reproducible production process is essential to preserve bacterial functionality and activity. Enhanced surface characterization of engineered bacteria can facilitate spatio-temporal specific colonization and functional expression in the inflammation sites, thereby improving the treatment efficacy. Precise dosimetry and modulation are also essential to enhance the efficacy and minimize the side effects. Additionally, genomic integration methods and biopreservation strategies are also needed to lower the risks of horizontal gene transfer (HGT) and ensure biosafety. In this review, the recent advancements of engineered bacteria and their derivatives as oral delivery systems for IBD therapy are highlighted, with a focus on surface coating modifications, genetic engineering, OMVs, minicells, and BGs. Their future prospects for clinical translation are also discussed (Figure 1).

### Pathological characteristics of IBD

**Figure 2:**
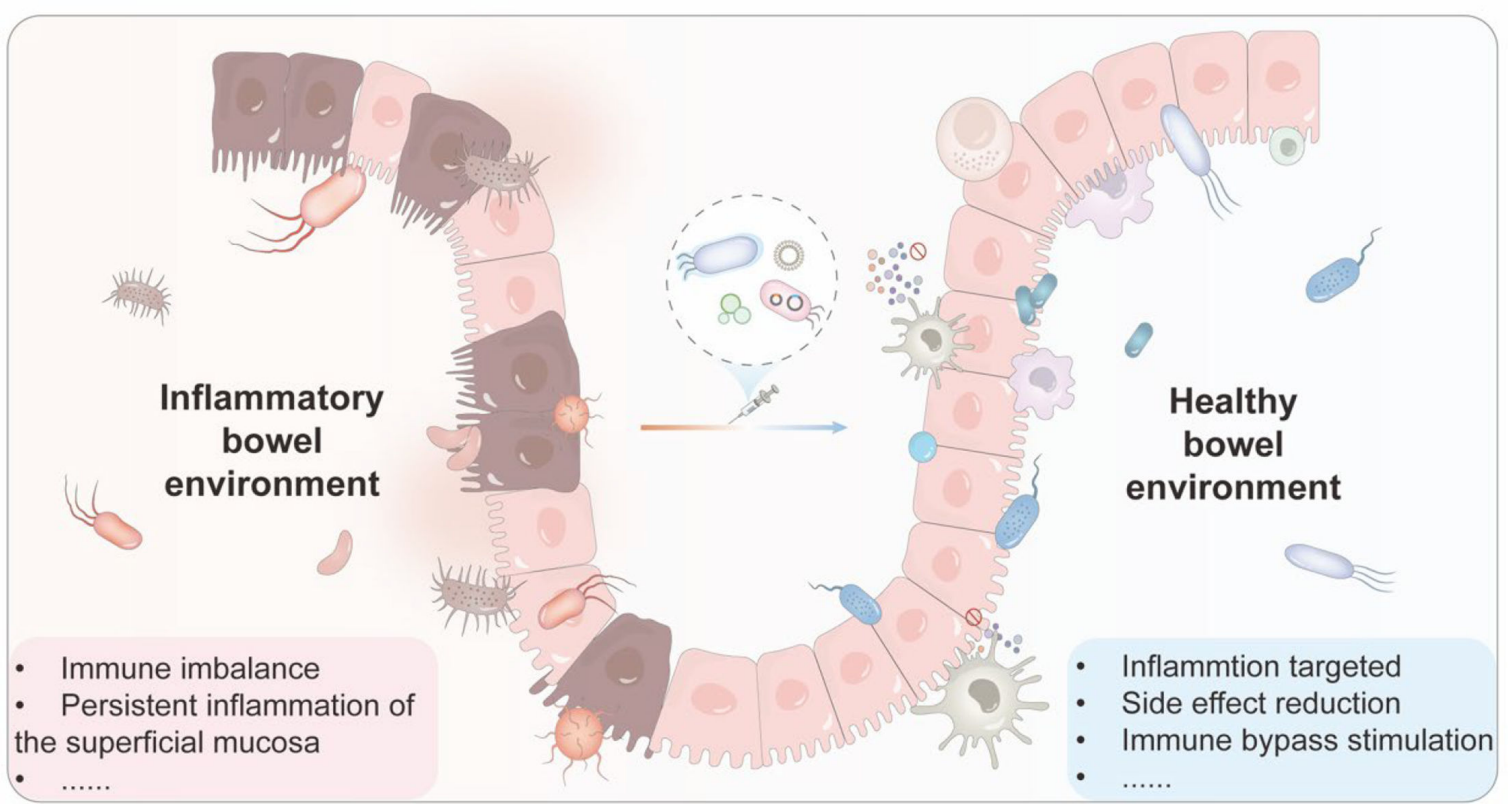
Engineered bacterial therapy enhances the efficacy of IBD treatment through a synergistic response of drug delivery and immune stimulation.

UC is localized to the colon, where it presents as continuous inflammation of the superficial mucosa, whereas CD can affect any segment of the gastrointestinal tract and is characterized by discontinuous transmural inflammation. The etiology of IBD is multifaceted, involving a combination of genetic, environmental, immunological, and intestinal microbial factors, all of which contribute to the predisposition and exacerbation of the disease. Studies have identified several gene loci associated with increased susceptibility to IBD, such as NOD2(nucleotide-binding oligomerization domain containing 2) and IL-23R (Interleukin-23 receptor) [29–31]. Additionally, pathogenic infections, dietary and other environmental factors, immune system dysregulation, and disturbances in gut microbiota all play a significant role in the initiation and progression of IBD [32,33]. Inflammatory biomarkers commonly associated with IBD include calprotectin, lactoferrin, reactive oxygen species (ROS), reactive nitrogen species, and hydrogen sulfide [34]. These mediators have a short half-life within the gut, contributing to the chronic cycle of relapse and remission [35]. Alongside conventional prescription therapies, over-the-counter probiotics play an essential role in repairing the intestinal mucosal barrier and modulating the immune rebalance [36]. Engineering bacterial therapies involve several mechanisms, including the neutralization of pro-inflammatory factors, regulation of specific immune cell subsets, blockade of signaling pathways, and modulation of lymphocyte migration (Figure 2). Consequently, the intelligent design of engineered probiotics is becoming increasingly critical for personalized medicine. This approach includes the development of inflammation-responsive drug delivery systems, controlled release-and-feedback regulations, and multifunctional integrated platforms. By tailoring therapeutic strategies according to the specific biochemical and physical characteristics of IBD, the long-term treatment outcome can be significantly improved [37].

### Applications and modification strategies of engineered bacteria and derivatives in IBD therapy

#### Chemical, physical, and biological coating

In oral bacterial delivery systems, chemical coatings play a crucial role in reducing direct exposure of bacteria to the external environment *in vitro*, which is essential for maintaining stability during long-term storage [38]. *In vivo*, these coatings serve a dual purpose: they not only shield bacteria from the harsh conditions of the gastrointestinal tract, such as the acidic condition and the presence of digestive enzymes, but also enable functional modifications that promote targeted delivery to specific sites of inflammation [39]. Several techniques are commonly employed to chemically modify bacterial surfaces, including covalent binding and *in situ* deposition [40]. For instance, natural polyphenols, through metal ion chelation, can rapidly form metal-polyphenol coordination complexes, a process that modifies engineered *Escherichia coli* Nissle1917 (EcN) and thus reshapes the immune microenvironment, providing therapeutic potential against IBD [41].

In addition to chemical coatings, physical coating strategies also play an integral role in enhancing bacterial delivery. These include electrostatic interactions, physical extrusion, and single or multilayer assembly. Electrostatic interactions rely on the negative charge on bacterial surfaces to attract positively charged nanoparticles, while physical extrusion encourages the self-coating of *Bacillus subtilis* with biofilm, enhancing bacterial colonization in the gut [42]. For example, self-assembled silk fibroin nanoparticles have been used to significantly increase bacterial survival under acidic conditions in the stomach and have shown enhanced therapeutic efficacy in mouse models of mucosal injury [43]. However, single-layer coatings, while effective in some cases, form physical barriers that limit the exchange of nutrients or metabolites, which may affect normal bacterial growth and proliferation. To alleviate this problem, multilayer assembly coatings have been developed. They can be degraded in the presence of external inflammatory stimuli, thereby facilitating controlled drug release and targeted therapy [44]. For example, the engineered EcN with a chitosan/sodium alginate coating was formed by layer-by-layer electrostatic self-assembly, which exhibited better protection in the gastrointestinal tract compared with eudragit L100-55, an enteric coating in clinically used drugs. The modified EcN showed rapid repairing effects in acute IBD syndromes by expressing catalase and superoxide dismutase [16].

Apart from chemical and physical coatings, biological modification is another important way to engineer bacteria. Glycoproteins on the surface of bacteria are key sites for linking and introducing exogenous enzymes, which are capable of neutralizing ROS. The ROS concentration is highly associated with intestinal inflammation in the IBD environment, suggesting the potential for biological surface modification in the treatment of IBD [45]. In addition, metabolic glycan engineering and azide-based metabolic labeling are also advanced strategies for further biomodification, offering new opportunities for broad bacterial functions [46]. Physical, chemical, and biological coating applied to bacteria and their derivatives can impart specific functions, such as promoting bacterial colonization, sustained-controlled release, and targeted therapy. Additionally, coatings can protect bacteria from physiological barriers, such as gastric acid, improving their effectiveness in treating IBD [16,47]. However, coatings also present some drawbacks. They may alter the characteristics of the bacteria and their derivatives, potentially affecting their activity and limiting the broader application.

#### Genetic engineering

*In situ* display on the bacterial surface or through biosynthesis of drugs showed efficacy *in vitro*. However, the main reason for limited treatment *in vivo* is that the yield is low and the difficulty in achieving therapeutic dose [34]. Despite the benefits of natural antioxidant enzymes in reducing inflammation, their activity is only exercised after bacterial lysis or rupture, limiting their effectiveness in their intact and active state [48]. In IBD inflammatory microenvironment, natural enzymes are easily and quickly inactivated, thus diminishing their therapeutic potential. One way to improve the efficacy is to design EcNs that can directly regulate intestinal immune function. EcNs can reduce inflammation by secreting anti-tumor necrosis factor-α (TNF-α) nanoantibodies, or by being engineered to express the anti-inflammatory protein AvCystatin [49,50]. Gene expression systems play an important role in this regard. On the one hand, constitutive expression systems continuously activate gene expression regardless of external stimuli in an independent manner. For example, modified strains of *Lactococcus lactis* secrete anti-inflammatory factors such as interleukin (IL)-10 or IL-27, which significantly attenuate inflammatory symptoms in a mouse model of colitis [51–53].

On the other hand, inducible expression systems provide a more controlled technique that can precisely promote and regulate gene transcription. The combined system relies on specific and external inducers, such as arabinose [54], xylose [55], xylan [56], or even light [57]. This design approach allows to regulate the release of therapeutic agents in response to specific environmental signals. A refined and sophisticated engineered system, such as the i-ROBOT platform, was introduced. The incorporated feedback control loop allowed the bacteria to sense biomarkers, diagnose the disease status, and release the corresponding therapeutic substances accordingly. This approach could control the release of therapeutic drugs based on specific environmental signals [58]. In another creative approach, engineered EcN strains were used to sense the fluctuations of molecules such as tetrathionate and nitric oxide (NO) in the intestinal environment [59]. Once detecting these metabolites, the bacteria released antimicrobial peptides and anti-inflammatory factors to reduce the inflammatory response [60]. In addition, bacteria designed to target specific metabolites secrete beneficial substances such as short-chain fatty acids and tryptophan, which are expected to modulate the host’s gut microbiota and metabolic activities [61,62]. This approach not only alleviates localized inflammation but also rebalances the gut microbiome, which is a key aspect of the pathogenesis of IBD.

#### Outer membrane vesicles

OMVs secreted by gram-negative bacteria have become an effective implement for both drug delivery and therapeutic intervention [63]. The host digestive tract possesses a complex and diverse microbial ecosystem that plays a crucial role in energy utilization, metabolism conversion, and immune regulation [64]. The microbial imbalance is closely associated with the onset and progression of IBD [65]. Nanoscale double-layered membrane particles’ OMVs offer several advantages in this context compared with conventional treatments [66]. The small size of OMVs allows them to effectively penetrate the mucosal barriers, including intestinal epithelial cell membranes and tissue interstitial junctions, thus enhancing the delivery of therapeutic agents to the target site [67]. Substantially, OMVs easily pass through the intestinal mucosal layer and prevent the cargo from degrading by the gastrointestinal enzymes, rendering them particularly attractive for oral drug delivery. By contrast, conventional approaches often encounter challenges in the protein hydrolysis environment [68].

OMVs have been modified to transport a wide variety of agents, such as immunomodulatory proteins, antibiotics, and enzymes, thus broadening their potential applications [69]. Beyond their drug delivery capability, OMVs convey bioactive signals interacting with immune cells, modulating the immune responses and potentially restoring immune homeostasis. For example, OMVs obtained from *Akkermansia muciniphila* have been applied to maintain the intestinal mucosal barrier, regulate gut microbiota, and stimulate an anti-inflammatory T-cell phenotype, leading to the reduction in IBD-related inflammation [70]. Likewise, OMV extracted from EcN can activate the NOD1 signaling pathway in intestinal epithelial cells and improve the expression of tight junction proteins that can enhance the barrier function, thereby further mitigating inflammation in IBD models [71,72].

In addition, OMVs obtained from *Bacteroides fragilis* can induce the differentiation of regulatory T cells (Tregs), thereby promoting IL-10 production through interaction with dendritic cells (DCs) by toll-like receptor 2 (TLR2) [73]. This action not only restrains colitis but also elevates mucosal tolerance, underscoring the modulatory potential of OMVs within the immune environment in IBD. Under this circumstance, OMVs from *B. fragilis* hold important therapeutic promise, especially for individuals with genetic defects that impair immune regulation [74]. In patients with ATG16L1 deficiency, these defects impair autophagy and Treg induction, but OMVs can bypass that by transmitting key immunomodulatory signals that promote Treg differentiation and inhibit proinflammatory responses. This ability to restore immune function reveals the therapeutic potential of OMVs for genetically susceptible individuals.

By bypassing the defective signaling pathway, OMVs offer a new, low-risk approach to reverse dysbiosis and re-establish immune tolerance. This gene-based treatment strategy addresses the underlying causes of IBD immune disorders. Therefore, OMV is not only expected to reduce inflammation but also offers a targeted solution for correcting immune system dysfunction. Their versatility offers an innovative option for genetically susceptible populations, with the potential to significantly impact the treatment landscape for IBD.

#### Minicells and bacterial ghosts

Compared with conventional therapies, minicells and BGs greatly diminish immunogenicity while preserving the therapeutic properties of parent bacteria [75]. Minicells are nanoscale bacteria with nuclei, which are often formed during abnormal cell divisions. They are naturally capable of wrapping and delivering therapeutics such as siRNA and small molecular drugs [76]. These minicells are usually induced by chemical stimulation and can therefore be precisely designed to target specific immune cells, particularly those in the intestinal adaptive immune system [77]. This chemically induced property renders them a precision drug delivery vehicle that can locate and regulate immune responses in the gut. In this context, a major drawback of traditional IBD antibiotic therapy is that HGT of resistance genes may occur during treatment, thus exacerbating the problem of bacterial resistance [78]. By contrast, the use of minicells to deliver therapeutic molecules is expected to reduce reliance on antibiotics and the risk of the spread of drug-resistant genes. The use of ligand-displaying minicells as antibiotic alternatives, which interact with the adaptive immune system, has the advantage of easy clearance *in vivo* while avoiding the accumulation of resistance genes [79]. The ability of minicells to both reduce immunogenicity and deliver therapeutic agents provides a promising avenue for minimizing adverse immune responses in IBD treatment.

On the other hand, BGs are inanimate bacterial envelopes that retain bacterial surface antigenicity, letting them attach effectively to the intestinal mucosa [80]. Unlike living bacteria, BGs are stripped of their metabolic activity but still retain their ability to interact with immune cells, making them excellent candidates for targeted drug delivery. For example, oral delivery of a mixture of three probiotic spore ghosts (*B. coagulans, B. subtilis,* and *B. licheniformis*) has been verified to restore intestinal mucosal damage. They coordinately work to rebalance the gut microbiota and reduce intestinal epithelial cell apoptosis [81]. Similarly, by inhibiting chemotaxis and ROS production in intestinal neutrophils, oral administration of EcN ghosts attenuates the inflammatory response in IBD [82].

Taken together, applications of minicells and BGs are new approaches to IBD treatment by providing smaller and genetically engineered vectors to deliver drugs and reduce systemic inflammatory responses. Their potential in targeted delivery, in particular their safety profile, underscores the promise of clinical application as a complementary strategy for IBD treatment where traditional therapies are inadequate (Table 1).

**Table 1:** Examples of bacterial engineering strategies

Engineering strategies	Modification	Bacteria	Delivery system	Refs
Chemical, physical, and biological coating	Chemical coating	*Escherichia coli Nissle1917*	EcN@SA-pBDT-TA	[41]
	Physical coating	*Bacillus subtilis*	Biofilm-coated BS (BCBS)	[42]
		*Escherichia coli Nissle1917*	SEcN4	[43]
		*Escherichia coli Nissle1917*	ECN-pE (C/A)2	[16]
		*Bifidobacterium longum*	SAzymes-armed probiotics BL@B-SA50	[45]
	Biological coating	*Consortia*	IgA-Consortia	[47]
Genetic engineering	/	*Escherichia coli Nissle1917*	PRObiotic type 3 secretion E. *coli* therapeutic (PROT3EcT)	[49]
		*Escherichia coli Nissle1917*	Transgenic EcN secreting A. viteae cystatin (EcN-AvCys)	[50]
		*Lactococcus lactis*	Thy12	[51]
		*Lactococcus lactis*	Genetically modified lactococcus lactis (LL-Thy12)	[52]
		*Lactococcus lactis*	LL-IL-27	[53]
Outer membrane vesicles	/	*Akkermansia muciniphila*	Akk OMVs	[70]
		*Escherichia coli Nissle1917*	ECOR12 OMVs	[71]
		*Escherichia coli Nissle1917*	EcN OMV	[72]
		*Bacteroides fragilis*	OMVs from B. fragilisΔpsa(ΔPSG-OMV)	[73]
		*Bacteroides fragilis*	Fragilis OMVs	[74]
Minicells and bacterial ghosts	Minicells	*Salmonella typhimurium*	Minicells packed with siRNA	[77]
	Minicells	*Escherichia coli Nissle1917*	Nb/Ag pairing modified bacteria	[79]
	Bacterial ghosts	*Streptavidin*	Streptavidin ghosts (SA-ghosts)	[80]
	Bacterial ghosts	*B.coagulans, B.subtilis and B.licheniformis*	BCSG, BSSG, and BLSG	[81]
	Bacterial ghosts	*Escherichia coli Nissle1917*	EcN ghosts	[82]

### Clinical challenges of engineered bacteria therapy in IBD treatment

Engineered bacteria hold great promise for the treatment of IBD, but their clinical application remains hampered by several challenges. One of the main concerns is that the functionality of engineered bacteria is closely linked to their surface properties, which directly influence their immunostimulatory capacity [83]. The dose-dependent nature of immune enhancement further complicates their use, particularly in immunocompromised IBD patients, where the response to such bacterial therapies may be weaker [84]. As a result, optimization of surface modifications and bacterial dosage is critical to improve the efficacy [85,86]. Another challenge is the possibility for engineered bacteria to start adverse reactions, such as sepsis and systemic inflammatory response syndrome, particularly if the bacteria spread uncontrollably [87,88]. Though many modified vectors are biocompatible and able to evade immune clearance, this ability increases the risk of unwanted proliferation and long-term toxicity [89]. As a result, effective infection management and precise selection of safer strains are considered to be able to minimize these risks. In addition, a key issue for therapeutic success is that the engineered bacteria may not express sufficient target genes *in vivo* [90]. To avoid that, it is essential to optimize the configuration of gene clusters, including the utilization of appropriate promoters and regulatory elements in order to regulate gene expression at specific times and locations. This guarantees an accurate and focused oral drug delivery, which can optimize the treatment outcomes. Furthermore, the introduction of foreign genes into bacterial vectors gives rise to concerns about the unintended introduction of toxic factors and antibiotic-resistance genes, which may compromise the therapeutic safety [91]. To mitigate these risks, it is imperative to integrate functional elements into the bacterial genome in a controlled manner or to implement bioprotective measures to prevent associated complications.

Overall, the clinical translation of engineered bacteria and bacterial derivatives for IBD treatment demands a well-designed delivery system. The system should theoretically combine precise signal conditioning and regulation, efficient processing, and secure output. By addressing the issues associated with immune stimulation, biosafety, gene expression accuracy, and gene transfer risk, engineered bacteria and bacterial derivatives could become valuable tools for the prevention, diagnosis, and treatment of IBD.

## Outlook and conclusion

This mini-review systematically provides an outline of the current engineering methodologies in using bacteria and their derivatives for IBD treatment, highlighting the obvious benefits of engineered bacterial strategies over traditional drug therapy. Engineered bacterial strategies offer significant benefits, such as precise targeting of inflammatory sites, modulation of the immune response, and minimizing adverse effects, all of which contribute to enhanced therapeutic outcomes. Despite significant progress, challenges remain, including the optimization of dosing regimens, overcoming immune escape mechanisms, and mitigating off-target gene effects. Nevertheless, engineered bacterial therapies hold significant potential for long-term benefits, including durable remission and the reduction in reliance on conventional drugs. To facilitate the use of engineered bacterial therapies for IBD, both the coating strategy and genetic engineering approach can be leveraged to maximize their therapeutic potential. Surface modifications, including chemical, physical, and biological coating, facilitate controlled drug release and targeted therapy. Researchers can also modify bacteria by knocking out or knocking down specific genes, which retain the bacteria’s essential functions while reducing pathogenicity, thereby improving the safety of their use in the gastrointestinal tract. Further advancements include the use of OMVs, minicells, and BGs, all of which allowed for specific immune stimulations in precision medicine. Personalized approaches, tailored to individual microbiome profiles and genetic predispositions, could further enhance the specificity and effectiveness of these therapies. Moreover, engineered bacteria could complement traditional treatments, optimizing overall therapeutic outcomes and offering more sustainable management of IBD. Key advantages encompass precise targeting of inflammatory sites, stimulation of adaptive immune responses, and the multifunctions for IBD therapy. However, before these strategies can be widely adopted in clinical settings, key challenges must be addressed, including improving the stability, delivery, and precision of genetically modified bacteria. Large-scale clinical trials will be essential for evaluating their safety, efficacy, and long-term impact. With a better understanding of bacterial structures, further research into new materials, and progress in modification techniques, engineered bacteria and bacterial derivatives are expected to become a more effective and sustainable approach to IBD management.

**Summary**

- The use of engineered bacteria and their derivatives in oral drug delivery systems holds significant clinical potential for treatment of inflammatory bowel disease. In particular, outer membrane vesicles can be used for immune bypass stimulation in patients with genetic defects, serving as an individualized design in precision medicine.
- To bridge theoretical research with clinical application, it is essential to refine modification strategies, including surface engineering and genetic design, with precise protocols for *in vivo* dosing, delivery frequency, and biosafety.
- Future advances in elucidating the biochemical and structural properties of these engineered bacteria and derivatives will be crucial to decipher host–microbial interactions within the gut and to identify novel therapeutic targets and the underlying mechanisms.

## Abbreviations

BGs: bacterial ghosts
CD: crohn's disease
DCs: dendritic cells
EcN: Escherichia coli Nissle1917
HGT: horizontal gene transfer
IBD: inflammatory bowel disease
IL-23R: interleukin-23 receptor
NO: nitric oxide
NOD2: nucleotide-binding oligomerization domain containing 2
OMVs: outer membrane vesicles
ROS: reactive oxygen species
TLR2: toll-like receptor 2
TNF-α: tumor necrosis factor-α
Tregs: regulatory T cells
UC: ulcerative colitis
